# Supplementary material for: Safety of the bag-in-the-lens implantation regarding the development of clinically significant pseudophakic cystoid macular edema: A retrospective case series study
Source: PLoS One. 2023 Jan 6;18(1):e0278861. doi: 10.1371/journal.pone.0278861 (PMC9821458; doi:10.1371/journal.pone.0278861)
Supplement: Attachment — Submitted filename: Rebuttal letter.docx [file pone.0278861.s002.docx]

04 April 2022

**Rebuttal letter – response to reviewers**

Dear reviewers, we revised our submission taking into account your constructive comments. We would like to thank you for your time for this exhaustive review.

- Please ensure that your manuscript meets PLOS ONE's style requirements, including those for file naming.
  - The manuscript was adapted according to the PLOS ONE style requirements.
- We noticed you have some minor occurrence of overlapping text with the following previous publication(s), which needs to be addressed.
  - We added a clear reference to the previous study of which this article is an extension/addition.
- Please clarify the sources of funding (financial or material support) for your study. List the grants or organizations that supported your study, including funding received from your institution. State what role the funders took in the study. If the funders had no role in your study, please state: “The funders had no role in study design, data collection and analysis, decision to publish, or preparation of the manuscript.”
  If any authors received a salary from any of your funders, please state which authors and which funders. If you did not receive any funding for this study, please state: “The authors received no specific funding for this work.”
  - The study was not supported by any external fund; the authors did not receive any specific funding for this work. We adjusted the disclosure and cover letter with more detailed information about study funding.
- In your Data Availability statement, you have not specified where the minimal data set underlying the results described in your manuscript can be found. PLOS defines a study's minimal data set as the underlying data used to reach the conclusions drawn in the manuscript and any additional data required to replicate the reported study findings in their entirety. All PLOS journals require that the minimal data set be made fully available.
  - The dataset on which our results are based was uploaded as Supporting Information.
  - The date of birth was removed from the database because this could, together with the date of surgery, compromise the patients’ anonymity.
- Although this study is a large case series, it states in its synopsis that “the rate of clinically significant pseudophakic cystoid macular edema after 'pocket-in-lens' implantation is comparable to traditional 'lens-in-bag'', indicating non-inferiority this safe surgical technique”, but this is only a retrospective non-comparative study and not a non-inferiority clinical trial. So it may not be appropriate to make this strong statement. I suggest changing the title to “Rate of clinically significant pseudophakic cystoid macular edema after 'pocket-in-lens' implantation: a retrospective case series study”.
- This is not a true non inferiority study in the sense that there is no control group, and the comparison is with the reports from the literature. A statement explaining this should be added to the limitations in the discussion, and accordingly the title of the study could be modified to reflect this.
  - The name of bag-in-the-lens is the official name of the IOL implantation technique as stated in the US and EP patent. As a result, we cannot change the name unless we use the inventor’s name “Tassignon IOL” as also stated in the patents (US and EPO). This is, however, not the authors’ first choice. We therefore will keep the “bag-in-the-lens” throughout the paper.
  - We would like to stress that the aim was not to compare the BIL with the LIB but to compare the CSPME after BIL with the incidence of CSPME after LIB as found in the literature. This is a big difference. If we would have done a comparative study, this would have shown the superiority of one technique compared to the other. We therefore deleted the term non-inferiority in the title but used the term non inferior compared with the incidence of CSPME in the LIB as described in literature.
  - We changed the title as suggested by the reviewer.
- The authors conclude that Primary Posterior Continuous Curvilinear Capsulorrhexis (PPCCC) does not increase the risk of CSPME. It would be prudent to add in this sentence the period (3 months) of postoperative interval.
  - We agree with the reviewers and add to the conclusion that we investigated the rate of CSPME up to 3 months postoperatively.
- Another weakness of this report is that the authors adopted "clinically significant pseudophakic cystoid macular edema". Therefore, postoperative optical coherence tomography (OCT) was not routinely performed in this retrospective study and was only implemented when requested by the attending physician.
- Under Methods; the authors explain how they defined pseudophakic clinically significant macular edema on OCT when it was ordered, but what is the definition used if no OCT was obtained. This should be clarified without ambiguity.
  - We examined the rate of clinically significant pseudophakic macular edema and therefore only performed OCT when there were complaints, and not routinely.
  - As soon as visual acuity decreased of 2 lines compared to the previous measurement or visual acuity did not improve postoperatively as expected preoperatively, an OCT was taken. Social security regulations recently changed the reimbursement criteria regarding preoperative OCT to cataract surgery. This regulation was, however, not in practice when our study was conducted. We do agree that this is a drawback in our study.
  - We only diagnosed CSPME after OCT was performed, which was ordered when visual acuity decreased. The combination of the changes on OCT and a significant subjective decrease in visual acuity was needed to conclude about CSPME.
- The authors expected CSPME to occur before the 3-month postoperative interval, but were the postoperative examinations performed at 1 day, 1 week, 5 weeks, 6 months, and one year postoperatively? How did they get the 3 month results? 50% were not followed up for at least 3 months postoperatively.
  - When visual acuity was 10/10, 5 weeks postoperatively, patients were requested to come back for sure in case of decreased visual acuity. If no return, we considered the situation unchanged.
- Allergy to diclofenac was not an exclusion criterion, so it is surprising that all patients used this drug for 4 weeks and no cases of intolerance were reported.
  - Diclofenac is used in our clinical center since its introduction. The cases of intolerance against diclofenac are extremely rare. This is the reason we do not consider this an exclusion criterion. We did, however, stop antibiotics in combination with corticosteroid drops already after 1 week to avoid confusion in intolerance between the different medication administered postoperatively.
- There are minor syntax errors in the manuscript that can be corrected with a final polish. (e.g. clinically significant instead of clinical significant in the Purpose section of the abstract; the word "after" is repeated twice in the Figure 2 Legend, etc...)
  - Some minor alterations were made.
- It would be prudent to add a sentence in the Methods section of the abstract to explain what are the risk factors studied in this cohort. The first mention of those is in the Results section of the abstract and this leaves the reader wondering about the meaning/implications.
  - This sentence was added in the Methods section.
- Under Introduction; line 4: the capsular bag approach is safer compared to previous generations of IOLs. The authors meant to compare implantation approaches rather than IOL designs, so this sentence should be revised.
  - This has been deleted.
- It is not clear what was the fate of cases operated using the described technique and had an inadvertent breech of the anterior hyaloid face. Where those cases excluded from the analysis? How many were there? How were they handled?
  - The bag-in-the-lens (BIL) is performed by very experienced surgeons. The incidence of anterior hyaloid breaches is very small if not existent. The BIL technique is erroneously considered as a difficult technique. It is based on the anatomical presence of the Berger space, which is in adult patients very big and in 60% of the patients older than 55 years the anterior hyaloid is already detached. This observation has recently been published in JCRS by A. Vael doi: 10.1097/j.jcrs.0000000000000866.

Many thanks for your comments,

Jasmien Rens and Prof. Dr. Marie-José Tassignon
