# Supplementary material for: Safety of the bag-in-the-lens implantation regarding the development of clinically significant pseudophakic cystoid macular edema: A retrospective case series study
Source: PLoS One. 2023 Jan 6;18(1):e0278861. doi: 10.1371/journal.pone.0278861 (PMC9821458; doi:10.1371/journal.pone.0278861)
Supplement: Attachment — Submitted filename: Rebuttal letter.docx [file pone.0278861.s003.docx]

01/11/2022

**Rebuttal letter – response to reviewers**

Dear editors and reviewers,

Thank you for your time in reviewing our paper in depth. Please find our reply to your comments:

- I would focus more on odds ratios additionally to the already used HR in the results section.
  - Adding odds ratios would mean adding a logistic regression model for the event rate over a certain period (e.g. 3 month). However, as not all patients had the same follow-up term, a Cox proportional hazards model was considered to be more appropriate to study the impact of risk factors on the occurrence of CSME/CSPME. In this way, patients with less than 3 months follow-up are not lost, as would be the case in a logistic regression model for the event rate at 3 months. The Cox proportional hazards model is summarized in hazard ratios instead of odds ratios, however interpretation is similar. The choice of the Cox model is further clarified in the paper.
- I would recommend to show the difference in central macular thickness (pre- to post-op) as a box plot.
  - The central retinal thickness was not included as a parameter in our statistical analysis. We only used the central retinal thickness in case of decline in CDVA to confirm the diagnosis of CSPME. As a result, pre- or post-op central macular thickness was not a parameter included in this paper.
- The study is well designed but the main draw back is retrospective nature of the study which makes us to conclude with caution. Retrospective study has many limitations and It will be defective in CME evaluation and recording specially when we have no OCT.
  - This limitation was already discussed, but the effect on drawing conclusions was added.

Based on these comments only a few minor additional adjustments have been made now. We submitted the manuscript with track changes.

Many thanks for your comments,

Jasmien Rens and Prof. Dr. Marie-José Tassignon
